# Dickkopf-1 Acts as a Profibrotic Mediator in Progressive Chronic Kidney Disease

**DOI:** 10.3390/ijms24087679

**Published:** 2023-04-21

**Authors:** Yung-Chien Hsu, Cheng-Chih Chang, Ching-Chuan Hsieh, Yu-Ting Huang, Ya-Hsueh Shih, Hsiu-Ching Chang, Pey-Jium Chang, Chun-Liang Lin

**Affiliations:** 1 Department of Nephrology, Chang Gung Memorial Hospital, Chiayi 613, Taiwan; libra@cgmh.org.tw (Y.-C.H.); ting94@cgmh.org.tw (Y.-T.H.); rita1608@gmail.com (Y.-H.S.); bettyleila529@gmail.com (H.-C.C.); 2 Kidney and Diabetic Complications Research Team (KDCRT), Chang Gung Memorial Hospital, Chiayi 613, Taiwan; 3 Department of Surgery, Chang Gung Memorial Hospital, Chiayi 613, Taiwan; m7021@cgmh.org.tw; 4 Division of General Surgery, Chang Gung Memorial Hospital, Chiayi 613, Taiwan; jeffrey570404@gmail.com; 5 Graduate Institute of Clinical Medical Sciences, College of Medicine, Chang Gung University, Taoyuan 333, Taiwan; 6 School of Traditional Chinese Medicine, College of Medicine, Chang Gung University, Taoyuan 333, Taiwan; 7 Kidney Research Center, Chang Gung Memorial Hospital, Taipei 105, Taiwan; 8 Center for Shockwave Medicine and Tissue Engineering, Chang Gung Memorial Hospital, Kaohsiung 833, Taiwan

**Keywords:** CKD, end-stage renal disease, DKK1, β-catenin, renal fibrosis

## Abstract

Chronic kidney disease (CKD) is a serious public health problem. Due to a high variability in the speed of CKD progression to end-stage renal disease (ESRD) and the critical involvement of Wnt/β-catenin signaling in CKD, we investigated the role of the Wnt antagonist Dickkopf-1 (DKK1) in CKD progression. Our data revealed that patients with CKD stages 4–5 had higher DKK1 levels in their serum and renal tissues than the control subjects. In an 8-year follow-up, the serum DKK1-high group in the enrolled CKD patients showed a faster progression to ESRD than the serum DKK1-low group. Using a rat model of 5/6 nephrectomy (Nx)-induced CKD, we consistently detected elevated serum levels and renal production of DKK1 in 5/6 Nx rats compared to sham-operated rats. Importantly, the knockdown of the DKK1 levels in the 5/6 Nx rats markedly attenuated the CKD-associated phenotypes. Mechanistically, we demonstrated that the treatment of mouse mesangial cells with recombinant DKK1 protein induced not only the production of multiple fibrogenic proteins, but also the expression of endogenous DKK1. Collectively, our findings suggest that DKK1 acts as a profibrotic mediator in CKD, and elevated levels of serum DKK1 may be an independent predictor of faster disease progression to ESRD in patients with advanced CKD.

## 1. Introduction

Chronic kidney disease (CKD) is emerging as a major health problem worldwide, which often leads to end-stage renal disease (ESRD) and is associated with increased mortality [1,2,3,4,5]. Several risk factors for CKD progression have been broadly proposed, including older age, diabetes, proteinuria, hypertension, hyperuricemia, obesity and lower hemoglobin level [6,7,8,9,10,11]. Despite extensive efforts to manage some of these risk factors, patients with advanced CKD (stages 4–5) ultimately progress to ESRD, albeit at different speeds among individuals. Therefore, finding more reliable predictors for CKD progression or potential therapeutic targets for CKD may help prevent or delay disease progression. 

The Wnt/β-catenin signaling pathway is a highly conserved pathway in metazoan animals [12,13,14,15,16,17]. In the Wnt/β-catenin signaling pathway, the Wnt proteins act as ligands binding to the receptor Frizzed (Fzd) and coreceptor low-density lipoprotein receptor-related proteins 5 and 6 (LRP5/6). The Wnt-Fzd-LRP5/6 trimeric complex subsequently triggers a signaling cascade that leads to β-catenin stabilization. After nuclear translocation, β-catenin interacts with the T cell factor (TCF)/lymphoid enhancer-binding factor (LEF) transcription factors and transcriptionally activates the expression of downstream target genes. Although the Wnt/β-catenin signaling is normally silenced in adult kidneys, this signaling can be reactivated after renal injury [15,18,19]. The transient activation of Wnt/β-catenin signaling is considered beneficial for the repair and regeneration of the kidneys after injury [20,21,22]. However, sustained activation of the Wnt/β-catenin signaling pathway is detrimental to the kidneys and contributes to renal fibrosis in CKD [23,24,25,26,27,28]. In this regard, several studies have reported that the blocking of specific Wnt/β-catenin signaling components by antagonists or chemical inhibitors could substantially ameliorate fibrotic CKD [23,24,25,26,27,29].

Various endogenous antagonists that block Wnt/β-catenin signaling have been found in vertebrates, including the Dikkopf (DKK) protein family, the secreted Frizzled-related protein (sFRP) family, Wnt inhibitory factor 1 (WIF-1), Klotho and sclerostin [30,31]. Among them, the DKK family proteins are secreted LRP5/6 antagonists that inhibit the formation of the Wnt-Fzd-LRP5/6 trimeric complex [30,32]. There are four DKK proteins (DKK1, DKK2, DKK3 and DKK4) identified in humans, and DKK1 appears to be the best characterized antagonist in the canonical Wnt/β-catenin signaling pathway [30,32]. In addition to LRP5/6, DKK1 may bind to other receptors such as Kremen1, Kremen2 or cytoskeleton-associated protein 4 (CKAP4) to produce diversified effects on cells [33,34,35,36]. Noteworthily, DKK1 is reported as a downstream target of Wnt/β-catenin signaling [37]; therefore, creating a negative feedback loop controlling Wnt/β-catenin signaling. To date, data on the role of DKK1 in CKD progression are scarce and inconclusive. In a mouse model of early CKD, Fang et al. showed that increased renal production and circulating levels of DKK1 were positively correlated with CKD pathogenesis [38]. Moreover, our previous studies revealed that the knockdown of DKK1 in streptozotocin-induced diabetic rats prevented renal dysfunction [39]. However, two other studies presented that delivering a DKK1 gene or recombinant DKK1 protein in a mouse model of unilateral ureteral obstruction (UUO) or adriamycin-induced nephropathy attenuated renal fibrosis [23,24]. Thus, the contribution of DKK1 to CKD progression needs further clarification.

In this study, we showed that patients with CKD stages 4–5 exhibited higher levels of DKK1 in their serum and renal tissues than non-CKD control subjects. In an 8-year follow-up study with 50 CKD patients (stages 4–5), we found that the patients with higher levels of serum DKK1 progressed more quickly to ESRD than the patients with lower levels of serum DKK1. The potential role of DKK1 toward fibrogenesis was further addressed in a rat model of 5/6 nephrectomy-induced CKD and in cultured mouse mesangial cells. Our results suggest that DKK1 is a profibrotic mediator in CKD progression. 

## 2. Results

### 2.1. High Levels of Serum DKK1 Are Associated with CKD Progression

**Figure 1:**
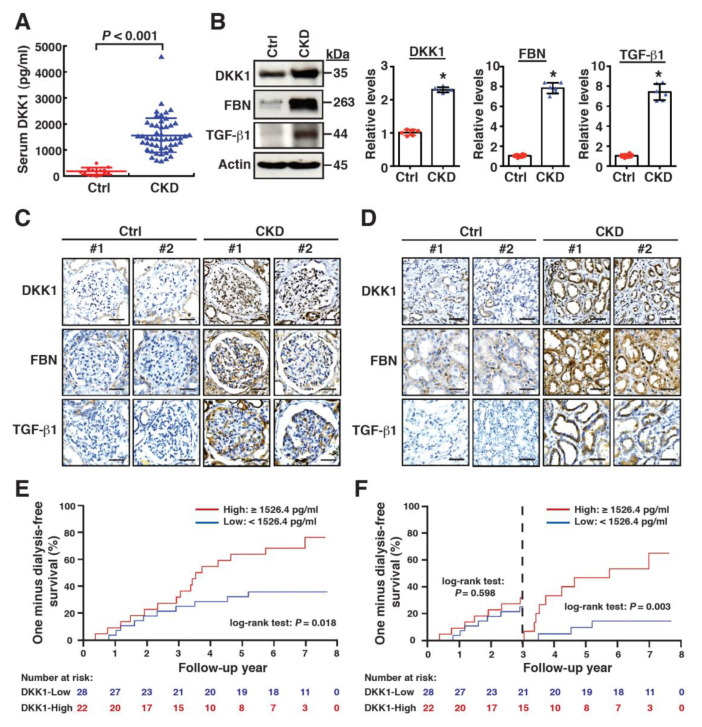
Elevated levels of serum DKK1 are associated with fast progression to ESRD in patients with CKD stages 4–5. (**A**) Serum DKK1 levels in normal controls (n = 15) and in patients with CKD stages 4–5 (n = 50). Data are presented as mean ± SD. (**B**) Western blot analysis for the expression of DKK1, fibronectin (FBN) and TGF-β1 in renal tissues from non-CKD and CKD subjects. Quantitative data are expressed as mean ± SD. * *p* < 0.05 versus control subjects (n = 6). (**C**) Chromogenic IHC staining for DKK1, FBN and TGF-β1 in renal glomeruli of representative non-CKD subjects (#1 and #2) and CKD subjects (#1 and #2). Scale bars: 20 μm. (**D**) Chromogenic IHC staining for DKK1, FBN and TGF-β1 in renal tubules of representative non-CKD subjects (#1 and #2) and CKD subjects (#1 and #2). Scale bars: 20 μm. (**E**) One minus dialysis-free survival analysis of CKD 4–5 patients with high serum levels of DKK1 (≥1526.4 pg/mL; n = 22) or low serum levels of DKK1 (<1526.4 pg/mL; n = 28) in an 8-year follow-up. (**F**) Stratification by 3-year follow-up in CKD 4–5 patients with low or high serum levels of DKK1.

Since active Wnt/β-catenin signaling is closely associated with CKD pathogenesis and DKK1 is an important secreted inhibitor of Wnt/β-catenin signaling, we compared the serum DKK1 levels between the predialysis patients with stages 4–5 CKD and the normal control subjects. The flow chart of CKD patients recruited in the study is shown in Supplementary Figure S1. The serum samples were then collected from 50 patients with CKD stages 4–5 and 15 normal control subjects. As shown in Figure 1A and Supplementary Table S1, the serum DKK1 levels were significantly higher in the CKD patients than in the normal control subjects (1559.4 ± 701.6 pg/mL versus 176.2 ± 132.2 pg/mL, *p* < 0.001). On the other hand, the Western blot analysis and immunohistochemical (IHC) analysis also revealed that the CKD patients had higher expression levels of DKK1, along with fibronectin and TGF-β1 in their kidney tissue samples compared to the non-CKD subjects (Figure 1B–D). These results suggested that the elevated serum levels and renal production of DKK1 might be associated with the pathogenesis or progression of CKD. To further evaluate the association between the serum DKK1 levels and CKD progression, the enrolled CKD patients (n = 50) were initially divided into two equal groups based on their serum DKK1 levels, namely, the DKK1-high group (≥1440.0 pg/mL, n = 25) and the DKK1-low group (<1440.0 pg/mL, n = 25). In an 8-year follow-up from 2008 to 2016, the DKK1-high group had a higher risk for faster progression to ESRD than the DKK1-low group (Supplementary Figure S2, *p* = 0.042). Using the receiver-operating characteristic (ROC) curve analysis, the optimal cutoff value of serum DKK1 was determined at 1526.4 pg/mL (Supplementary Figure S3; area under curve 0.673, 95% confidence interval 0.518–0.828, sensitivity 61.5% and specificity 79.2%). Based on the optimal cutoff level, the CKD patients were regrouped as the DKK1-high group (≥1526.4 pg/mL, n = 22) and the DKK1-low group (<1526.4 pg/mL, n = 28) (Supplementary Table S1). The Kaplan–Meier analysis of dialysis-free survival showed that the DKK1-high group progressed more quickly to ESRD than the DKK1-low group (Figure 1E, *p* = 0.018). Noteworthily, a marked divergence in the dialysis-free survival curves of the two groups appeared after the 3-year follow-up (Figure 1F, *p* = 0.598 in years 0–3; *p* = 0.003 in years 3–8).

Table 1 shows the comparison of the baseline demographic characteristics and biochemical variables between the DKK1-high and DKK1-low groups of CKD patients. Except for the daily urine output (*p* = 0.015), there were no statistically significant differences in the demographic characteristics and biochemical parameters between the two groups (Table 1). After adjusting for age, sex, baseline eGFR, hemoglobin, albumin, high-sensitivity C-reactive protein and daily urine output, our results revealed that the high serum DKK1 levels (per 100 pg/mL increase) were associated with an increased risk of kidney failure in the CKD patients (Table 2). However, there was no significant association between the serum DKK1 levels and the risk of all-cause mortality (Table 2).

### 2.2. Treatment with DKK1-Antisense Oligonucleotides Attenuates CKD-Associated Phenotypes in a 5/6-Nephrectomized Rat Model

**Figure 2:**
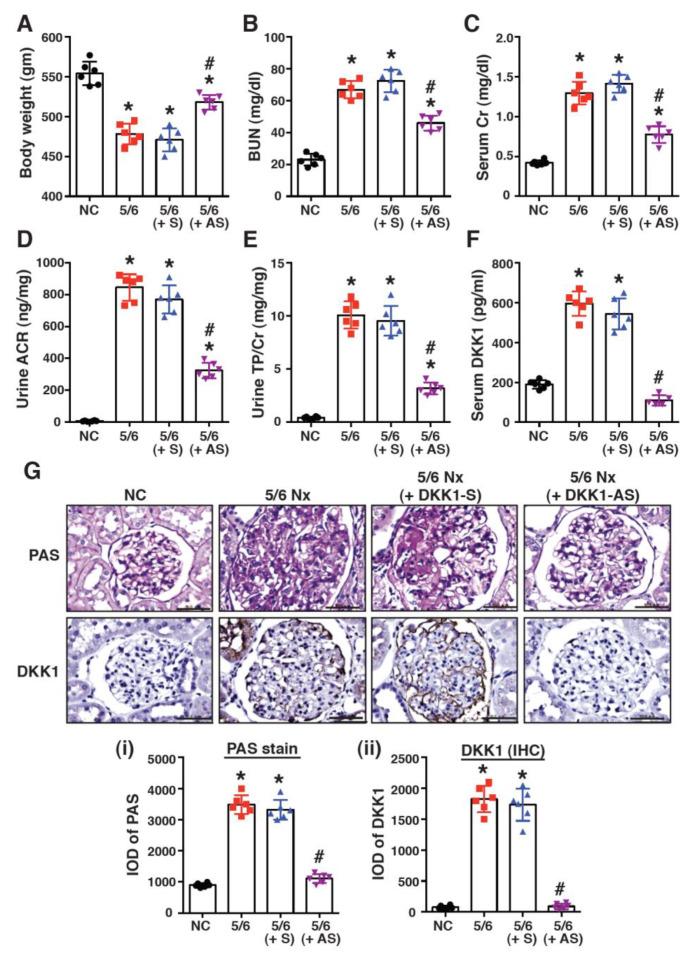
Treatment of 5/6-nephrectomized (5/6 Nx) rats with the DKK1-antisense (DKK1-AS) oligonucleotide alleviates CKD-associated phenotypes. Body weight (**A**), blood urea nitrogen (BUN) level (**B**), serum creatinine (Cr) level (**C**), urine albumin/creatinine ratio (ACR) (**D**), urine total protein/creatinine ratio (TP/Cr) (**E**) and serum DKK1 level (**F**) were analyzed in the sham-operated rats (NC) and in the 5/6 Nx rats that were left untreated or treated with the DKK1-sense (DKK1-S) or DKK1-antisense (DKK1-AS) oligonucleotide. Data are expressed as mean ± SD (n = 6 for each group). * *p* < 0.01 versus the sham group, # *p* < 0.01 versus the untreated 5/6 Nx group. (**G**) Representative images of PAS staining and IHC staining for DKK1 in renal glomeruli of the indicated treated groups. Scale bars: 50 μm. Intensities of PAS staining and IHC staining for DKK1 in kidney sections from different groups were determined by quantitative integrated optical density (IOD) analysis and plotted in panels (**i**) and (**ii**), respectively. Data are represented as mean ± SD (n = 6 for each group). * *p* < 0.05 versus the sham group, # *p* < 0.05 versus the untreated 5/6 Nx group.

To investigate the potential role of DKK1 in CKD, a 5/6-nephrectomized (5/6 Nx) rat model was used. As compared to sham-operated rats, the mean body weight was significantly reduced in the 5/6 Nx rats (Figure 2A, *p* < 0.01). Moreover, the 5/6 Nx rats exhibited elevated levels of blood urea nitrogen (BUN), creatinine (Cr), albuminuria (urine albumin/creatinine ratio, ACR) and proteinuria (urine total protein/creatinine ratio, TP/Cr), in comparison with the sham-operated rats (Figure 2B–E, *p* < 0.01). Serum DKK1 was also detected at higher levels in the 5/6 Nx group compared to the sham group (Figure 2F, *p* < 0.01). In parallel experiments, the 5/6 Nx rats were treated with the DKK1-antisense (DKK1-AS) or the DKK1-sense oligonucleotide (DKK1-S). After 8-weeks of treatment, only DKK1-AS, but not DKK1-S, reduced the serum DKK1 levels in the 5/6 Nx rats (Figure 2F, *p* < 0.01). Importantly, the DKK1-AS treatment also restored body weight and reduced the levels of BUN, Cr, ACR and TP/Cr in the 5/6 Nx rats (Figure 2A–E, *p* < 0.01). To further determine the association between DKK1 levels and renal fibrosis, kidney sections from the different treatment groups were stained with PAS or immunostained with an anti-DKK1 antibody (Figure 2G). The PAS stain showed that a pronounced accumulation of fibrotic matrix was observed in the glomeruli of the 5/6 Nx rats; however, the treatment with DKK1-AS (but not DKK1-S) substantially reduced the deposition of the fibrotic matrix in the glomeruli in the 5/6 Nx rats (Figure 2G). Based on the IHC staining results, we found that the expression levels of DKK1 in the glomeruli were positively correlated with renal fibrosis (Figure 2G). 

**Figure 3:**
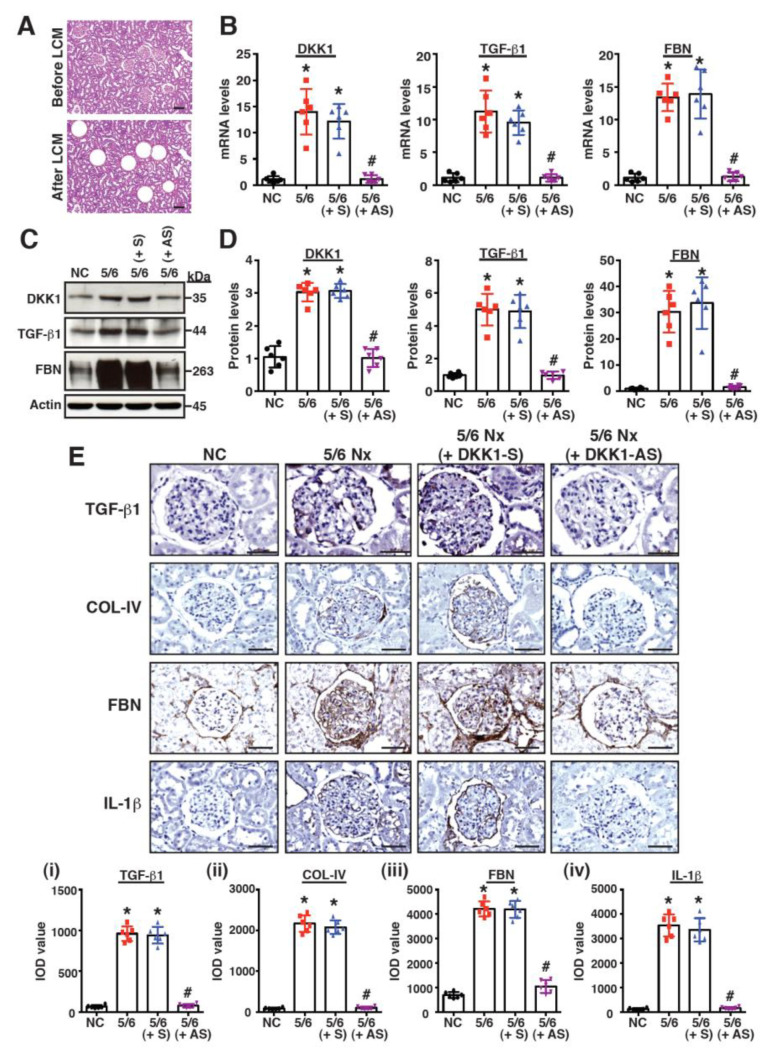
Evaluation of the expression levels of profibrotic and proinflammatory factors in renal tissues of the sham-operated rats and the 5/6 Nx rats left untreated or treated with DKK1-S or DKK-AS. (**A**) Representative photographs of renal sections before and after capturing glomerular compartments using laser capture microdissection (LCM). Scale bars: 100 μm. (**B**) Quantitative RT-PCR analysis of DKK1, TGF-β1 and fibronectin (FBN) mRNAs in isolated glomeruli microdissected from kidney sections of different groups. Quantitative data are represented as mean ± SD (n = 6 for each group). * *p* < 0.05 versus the sham group, # *p* < 0.05 versus the untreated 5/6 Nx group. (**C**) Representative images of Western blot analysis for the expression of DKK1, β-catenin, TGF-β1 and FBN in renal tissues of different groups. (**D**) Quantitative analysis of Western blots for DKK1, β-catenin, TGF-β1 and FBN in renal tissues of different groups. Data are plotted as mean ± SD (n = 6 for each group). * *p* < 0.05 versus the sham group, # *p* < 0.05 versus the untreated 5/6 Nx group. (**E**) IHC staining for TGF-β1, collagen IV (COL-IV), fibronectin (FBN) and IL-1β in renal glomeruli of different treated groups. Scale bars: 50 μm. Bottom panels show quantitative IOD analysis of the intensities of TGF-β1 (**i**), COL-IV (**ii**), FBN (**iii**) and IL-1β (**iv**) in renal glomeruli of different groups (n = 6 for each group). Data are represented as mean ± SD. * *p* < 0.05 versus the sham group, # *p* < 0.05 versus the untreated 5/6 Nx group.

In addition, the mRNA expression levels of DKK1 and the specific profibrotic factors in the renal glomeruli isolated with laser capture microdissection (Figure 3A) were evaluated using RT-qPCR. Compared to the sham group, the mRNA levels in DKK1, TGF-β1 and fibronectin were noticeably elevated in the 5/6 Nx group (Figure 3B). However, the treatment of the 5/6 Nx rats with DKK1-AS markedly diminished the levels of DKK1, TGF-β1 and fibronectin mRNAs in the glomeruli (Figure 3B). Similar results were also obtained from the Western blot analysis for the expression of DKK1, TGF-β1 and fibronectin in the renal tissues of the different treatment groups (Figure 3C,D). The IHC analysis further confirmed that both profibrotic and proinflammatory factors, such as TGF-β1, collagen IV, fibronectin and IL-1β, were highly expressed in the glomeruli of the 5/6 Nx group; however, these factors were reduced in the 5/6 Nx rats receiving the DKK1-AS treatment (Figure 3E). All of these results suggested that the elevated levels of DKK1 in the glomeruli were associated with renal fibrosis.

### 2.3. Increased DKK1 Expression Correlates with Accumulation of β-Catenin in Renal Tissues of 5/6 Nx Rats

**Figure 4:**
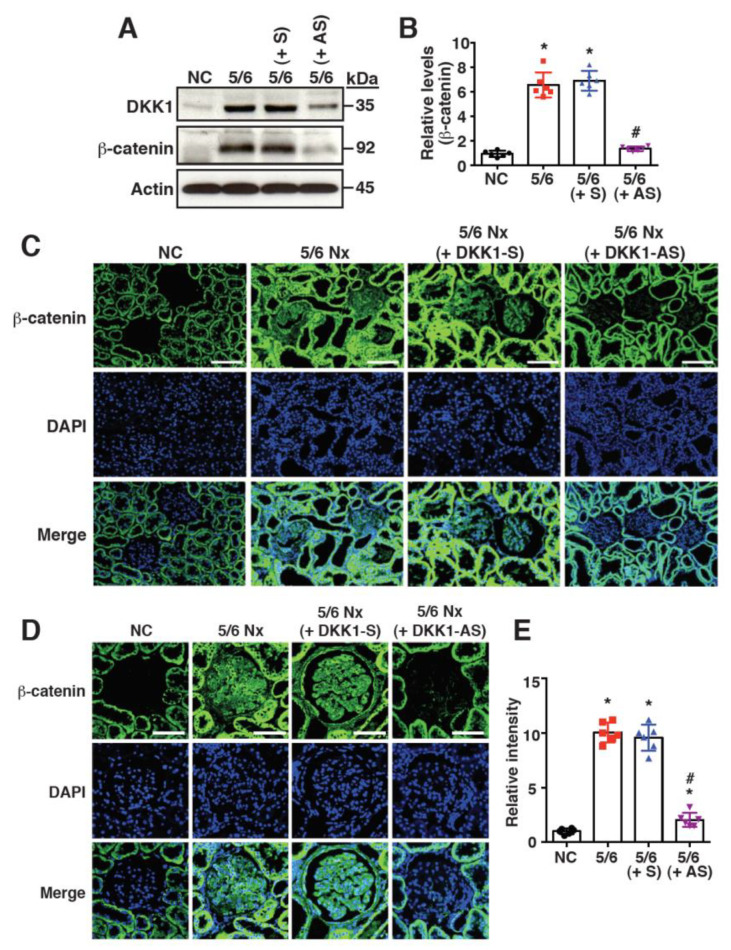
The renal expression of DKK1 positively correlates with accumulation of β-catenin in 5/6 Nx rats. (**A**) Representative images of Western blot analysis for the expression of DKK1 and β-catenin in renal tissues of the sham group and the 5/6 Nx groups that were untreated or treated with DKK1-S or DKK1-AS. (**B**) Quantitative analysis of Western blots for β-catenin in renal tissues of different groups (n = 6 for each group). * *p* < 0.05 versus the sham group, # *p* < 0.05 versus the untreated 5/6 Nx group. (**C**) Immunofluorescence analysis of β-catenin in kidney sections from the sham-operated rats and the 5/6 Nx rats left untreated or treated with DKK1-S or DKK-AS. Scale bars: 100 μm. (**D**) Immunofluorescence images of renal glomeruli stained for β-catenin in different treated groups. Scale bars: 50 μm. (**E**) Quantification of relative fluorescence intensities for β-catenin in renal glomeruli of different groups (n = 6 for each group). * *p* < 0.05 versus the sham group, # *p* < 0.05 versus the untreated 5/6 Nx group.

Since DKK1 is considered as an inhibitor of the Wnt/β-catenin signaling pathway, the expression of β-catenin was evaluated in the renal tissues of the different treatment groups. The Western blot analysis showed that both DKK1 and β-catenin were expressed at higher levels in the 5/6 Nx group than in the sham group (Figure 4A,B). Unexpectedly, the knockdown of DKK1 in the renal tissues of the 5/6 Nx rats using the DKK1-AS oligonucleotide led to a reduced β-catenin level (Figure 4A,B). The immunofluorescence analysis confirmed that cytoplasmic/nuclear β-catenin was abundantly accumulated in the renal glomeruli of the 5/6 Nx rats (Figure 4C–E); however, the treatment with DKK1-AS reduced the β-catenin accumulation in the renal glomeruli (Figure 4C–E). These results suggested that DKK1 might positively regulate β-catenin accumulation in the renal tissues of 5/6 Nx rats. 

### 2.4. Uremic Serum Stimulates Fibrogenesis of Mouse Mesangial Cells, at Least Partly, through DKK1-Mediated Signaling

**Figure 5:**
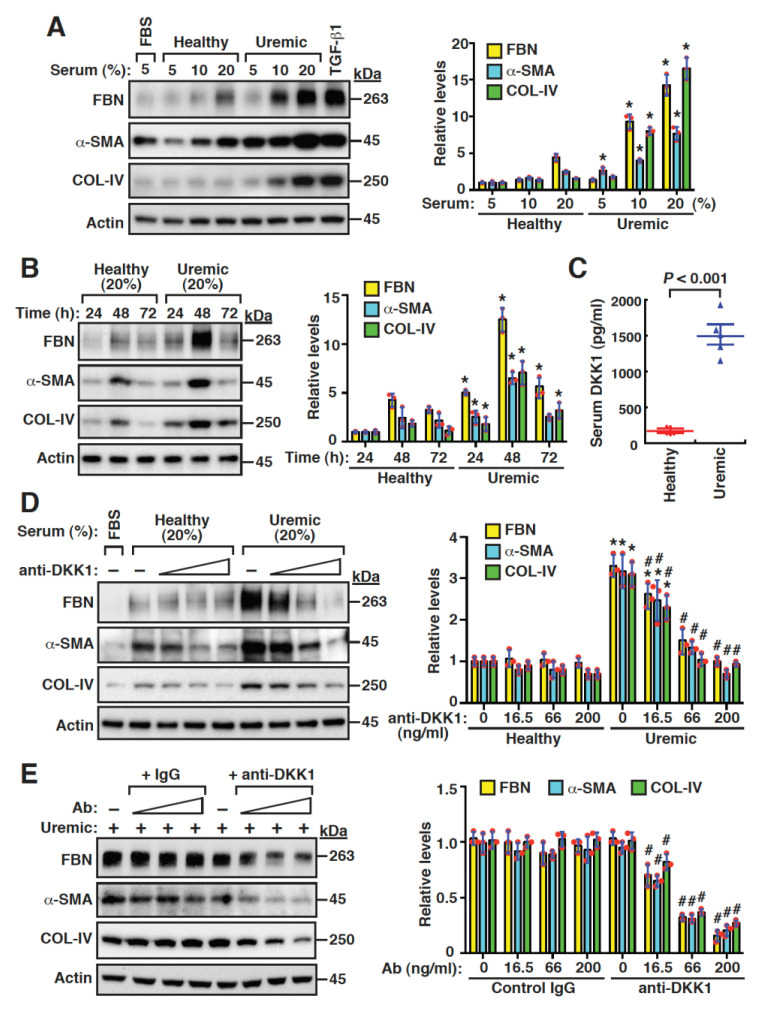
Uremic serum promotes fibrogenesis of renal mesangial cells through the DKK1-mediated signaling pathway. (**A**) Western blot analysis of fibronectin (FBN), α-smooth muscle actin (α-SMA), and collagen IV (COL-IV) expressed in mouse mesangial cells that were cultured in media with increasing concentrations (5, 10 and 20%) of healthy control serum or uremic serum. The healthy serum and uremic serum samples were pooled at equal volumes from blood samples of five healthy control subjects and five uremic patients, respectively. Quantitative data from Western blots are plotted as mean ± SD (n = 3). * *p* < 0.05 versus the healthy controls at the corresponding serum concentrations. (**B**) Western blot analysis of FBN, α-SMA and COL-IV expressed in mouse mesangial cells cultured in media with 20% healthy control serum or uremic serum for 24, 48 and 72 h. Quantitative data from Western blots are plotted as mean ± SD (n = 3). * *p* < 0.05 versus the healthy controls at the corresponding time points. (**C**) Serum DKK1 levels of five healthy control subjects and five uremic patients included in the in vitro experiments. (**D**) Effects of increasing amounts of anti-DKK1 neutralizing antibody (16.5, 66 and 200 ng/mL) on the expression of FBN, α-SMA and COL-IV in mesangial cells cultured in media with 20% healthy control serum or uremic serum. Quantitative data from Western blots are plotted as mean ± SD (n = 3). * *p* < 0.05 versus the healthy controls, # *p* < 0.05 versus the untreated uremic serum group. (**E**) Effects of increasing amounts of an IgG control and anti-DKK1 antibody on the expression of profibrotic proteins in mesangial cells cultured in media with 20% uremic serum. Quantitative data from Western blots are plotted as mean ± SD (n = 3). # *p* < 0.05 versus the untreated uremic serum group.

To investigate the potential role of serum DKK1 in the fibrogenic activation of mesangial cells, we initially cultured mouse mesangial cells in media with increasing concentrations (5, 10 and 20%) of healthy or uremic serum samples (pooled from five healthy donors or five uremic patients). The Western blot analysis showed that the uremic serum at 10 or 20% had greater promoting effects on the expression of fibronectin, α-smooth muscle actin (α-SMA) and collagen IV, in comparison with the healthy serum at the same concentrations (Figure 5A). The time-course experiments also demonstrated that the uremic serum induced higher levels of fibronectin, α-SMA and collagen IV than the healthy serum (Figure 5B). When the levels of serum DKK1 were measured, we indeed found that these levels were higher in uremic patients than those in the healthy controls (Figure 5C, 1496.0 ± 290.4 pg/mL versus 170.9 ± 49.1 pg/mL, *p* < 0.001). We then added an anti-DKK1 neutralizing antibody (16.5, 66.0 and 200 ng/mL) to the cultured mesangial cells. The addition of the anti-DKK1 neutralizing antibody to the healthy serum-cultured mesangial cells did not significantly affect the expression of fibronectin, α-SMA and collagen IV (Figure 5D). However, the levels of these profibrotic proteins in the uremic serum-cultured mesangial cells were markedly inhibited by the anti-DKK1 neutralizing antibody (Figure 5D). Furthermore, only the anti-DKK1 neutralizing antibody, but not the control antibody, specifically blocked the profibrotic protein expression induced by the uremic serum in the mesangial cells (Figure 5E). Overall, our results suggested that serum DKK1 might play a critical role in the fibrogenic activation of mesangial cells.

### 2.5. DKK1 Is a Profibrotic Mediator and Can Reinforce Its Own Expression in Mesangial Cells

**Figure 6:**
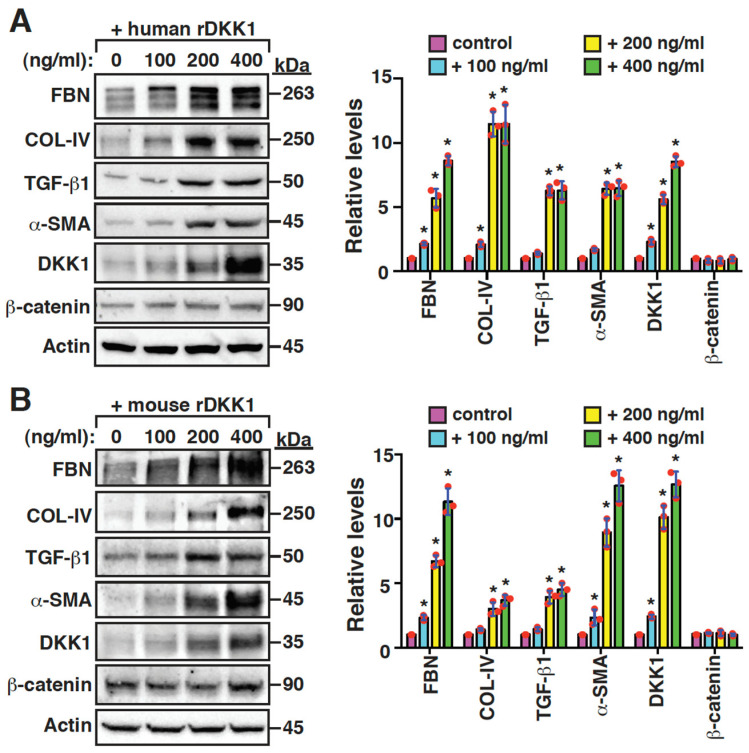

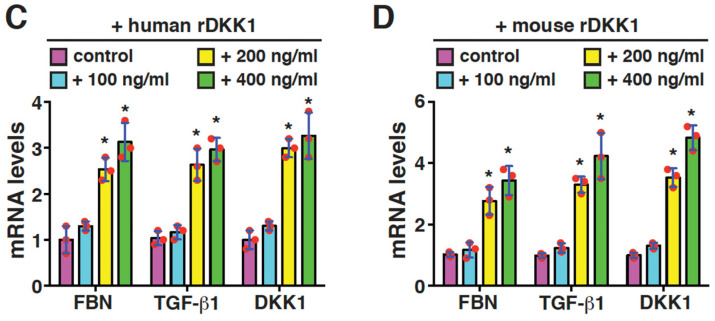
DKK1 acts as a profibrotic mediator in mouse mesangial cells. (**A**) Effects of increasing amounts of recombinant human DKK1 (100, 200 and 400 ng/mL) on the expression of FBN, COL-IV, TGF-β1, α-SMA, DKK1 and β-catenin in mouse mesangial cells. The quantitative results from Western blots are plotted as mean ± SD (n = 3). * *p* < 0.05 versus the untreated control. (**B**) Effects of increasing amounts of recombinant mouse DKK1 (100, 200 and 400 ng/mL) on the expression of the indicated proteins in mouse mesangial cells. The quantitative data from Western blots are plotted as mean ± SD (n = 3). * *p* < 0.05 versus the untreated control. (**C**) Quantitative RT-PCR analysis of FBN, TGF-β1 and DKK1 mRNAs expressed in mouse mesangial cells that were left untreated or treated with increasing amounts of recombinant human DKK1 (100, 200 and 400 ng/mL). * *p* < 0.05 versus the untreated control (n = 3). (**D**) Quantitative RT-PCR analysis of FBN, TGF-β1 and DKK1 mRNAs expressed in mouse mesangial cells that were left untreated or treated with increasing amounts of recombinant mouse DKK1 (100, 200 and 400 ng/mL). * *p* < 0.05 versus the untreated control (n = 3).

To further explore the role of DKK1 in promoting mesangial cell fibrogenesis, recombinant DKK1 proteins were added to the cultured mouse mesangial cells. The addition of either human DKK1 or mouse DKK1 significantly enhanced the expression of fibronectin, collagen IV, TGF-β1 and α-SMA (Figure 6A,B), supporting the finding that DKK1 is a profibrotic mediator. In contrast, neither human DKK1 nor mouse DKK1 influenced the overall levels of β-catenin in the mouse mesangial cells (Figure 6A,B). In these experiments, we additionally noticed that recombinant DKK1 proteins could induce the expression of endogenous DKK1 in a dose-dependent manner (Figure 6A,B). To further confirm our findings, the mRNA expression levels of DKK1 and different profibrotic factors were measured using RT-qPCR (Figure 6C,D). Our results demonstrated that the high concentrations (200 and 400 ng/mL) of recombinant human or mouse DKK1 protein were able to increase the mRNA levels of DKK1, fibronectin and TGF-β1 in the mesangial cells (Figure 6C,D). These findings suggested that circulating DKK1 might trigger a positive feedback loop to modulate the fibrogenesis of mesangial cells.

## 3. Discussion

In this report, we showed that the serum DKK1 levels were significantly higher in patients with advanced CKD than in the normal controls, and that the CKD patients with higher serum DKK1 levels progressed more quickly to ESRD. We therefore proposed that serum DKK1 could be an independent predictor for fast progression to ESRD in CKD patients. Currently, there is limited literature concerning the comparison of serum DKK1 levels between CKD patients and normal controls. Fang et al. [38] previously reported that increased serum DKK1 levels were detected in CKD patients and CKD mice. However, two other studies showed that CKD patients had lower serum DKK1 levels than non-CKD controls [40,41]. These controversial results could be explained by several factors including small sample sizes and the possible recruitment of some non-CKD controls that might have other conditions indirectly affecting the serum DKK1 levels. Although there were no conclusive data on the serum DKK1 levels in CKD and non-CKD subjects, our studies clearly showed that high levels of serum DKK1 in the CKD patients were associated with faster progression to ESRD in an 8-year follow-up (Figure 1E). Intriguingly, we noticed here that within the first 3-year follow-up, the dialysis-free survival curves did not significantly differ between the DKK1-high and DKK1-low groups (Figure 1F). These findings suggested that multiple risk factors could be heavily accumulated in the DKK1-high group within the first 3 years of CKD stage 4 or 5 to make a sudden change in kidney function. 

In addition to dialysis initiation, the association of serum DKK1 level with all-cause mortality in the enrolled CKD patients was also evaluated in the study. However, we did not find a significant association between the serum DKK1 levels and the risk of all-cause mortality (Table 2). In the 8-year follow-up, 5 of the 22 patients with high levels of serum DKK1 (≥1526.4 pg/mL) were dead (22.7%), whereas 7 of the 28 patients with low levels of DKK1 (<1526.4 pg/mL) were dead (25.0%). The mortality rates between the serum DKK1-high and DKK1-low groups were not significantly different. The most common causes of death were sepsis (four for the DKK1-high group; three for the DKK1-low group) and cardiovascular diseases (one for the DKK1-high group; four for the DKK1-low group). Due to the small sample size in the present study, further studies with larger samples sizes may be required to obtain supportive conclusions.

Although active Wnt/β-catenin signaling is commonly associated with CKD, the expression level and functional role of DKK1 in CKD remain obscure. In a mouse model of early CKD, Fang et al. showed that CKD mice produced higher levels of serum DKK1 than non-CKD mice [38]. Furthermore, He et al. reported that DKK1 was upregulated in the renal tissues of UUO mice [23]. Consistent with these previous observations, we found here that DKK1 was detected at higher levels in the serum and renal tissues of 5/6 Nx rats as compared to sham-operated rats (Figure 2 and Figure 3). In particular, DKK1 expression in the renal tissues of the 5/6 Nx rats was strongly correlated with renal fibrosis (Figure 2 and Figure 3), and the knockdown of DKK1 by DKK1-AS remarkably attenuated the CKD-associated phenotypes (Figure 2 and Figure 3). These results suggested that DKK1 was not only the biomarker of fibrotic CKD, but also a key effector of renal fibrosis.

**Figure 7:**
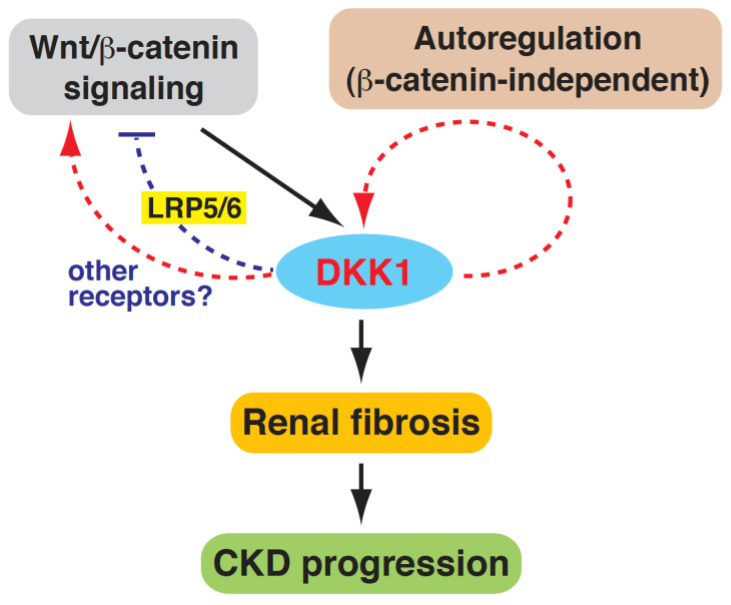
Proposed model of DKK1-mediated renal fibrosis. During renal injury, DKK1 can be initially activated by the Wnt/β-catenin signaling pathway. Subsequently, DKK1 may autoregulate its own expression to further increase its levels. The DKK1 autoregulation may be unrelated to the Wnt/β-catenin signaling pathway. Increased expression of DKK1 is involved in renal fibrosis and thus promotes CKD progression. Under the circumstances of renal injury, DKK1 is no longer acting as a Wnt/β-catenin antagonist (via interaction with LRP5/6). Conversely, DKK1 may activate the Wnt/β-catenin signaling by some unknown mechanisms in renal cells or in infiltrating immune cells.

Intriguingly, although DKK1 is considered as an inhibitor of Wnt/β-catenin signaling, we found here that the levels of both DKK1 and β-catenin were abundantly expressed in the 5/6 Nx rats compared to the sham-operated rats. Since DKK1 is known as a direct target in Wnt/β-catenin signaling, it is possible that active Wnt/β-catenin signaling induced by subtotal 5/6 nephrectomy may augment the DKK1 expression. The secreted DKK1 may subsequently inhibit the Wnt/β-catenin signaling through binding to LRP5/6 (Figure 7). However, we found that the knockdown of DKK1 in the 5/6 Nx rats led to a significant reduction in the β-catenin accumulation in the renal tissues (Figure 4). Our results indicated that under the circumstances of 5/6 Nx-induced CKD, DKK1 was no longer functioning as a Wnt/β-catenin antagonist, but it might act as a Wnt/β-catenin activator. Due to the complicated involvements of different renal cell types and infiltrating immune cells in renal fibrosis, we propose that DKK1 may positively regulate β-catenin accumulation by dynamic cell-cell activities via some unknown mechanisms (Figure 7). Additionally, DKK1 may positively activate the Wnt/β-catenin signaling pathway in renal cells through specific receptors such as CKAP4, but not LRP5/6 (Figure 7). This is not so surprising because several studies have revealed that both DKK1 and β-catenin could be abundantly detected in a variety of human cancers such as chondrosarcoma and hepatocellular carcinoma (HCC) [32,42,43,44,45]. Moreover, Zhang et al. showed that DKK1 could promote the proliferation and tumorigenicity of HCC cells via activating the Wnt/β-catenin signaling pathway [44].

For characterizing the profibrogenic effect of DKK1 on renal cells, our studies showed that uremic serum could stimulate the fibrogenesis of mouse mesangial cells, at least in part, through the DKK1-mediated signaling pathway (Figure 5). We additionally found that recombinant DKK1 protein was sufficient to induce the expression of multiple profibrotic factors in cultured mesangial cells (Figure 6), supporting that DKK1 was a profibrotic mediator. Intriguingly, recombinant DKK1 protein not only stimulated the production of the well-known profibrotic proteins, but also activated the expression of endogenous DKK1 in cultured mesangial cells (Figure 6). Since we did not find a significant change in β-catenin accumulation in the cultured mesangial cells after exposure to recombinant DKK1 protein (Figure 6), the DKK1 autoregulation appeared to be unrelated to the Wnt/β-catenin signaling pathway (Figure 7). In future research, the detailed molecular mechanism underlying DKK1 autoregulation and the association of DKK1 levels with β-catenin accumulation in mesangial cells need to be further investigated. In addition to glomerular mesangial cells, the DKK1-mediated signaling may play an important role in different renal cell types, especially tubular epithelial cells, to promote renal fibrosis (tubulointerstial fibrosis). Further studies are warranted to investigate the profibrotic role of DKK1 in tubular epithelial cells. 

## 4. Materials and Methods

### 4.1. Study Design and Population

This cohort study enrolled predialyis patients with stage 4 or 5 CKD (estimated glomerular filtration rate (eGFR): 5–29 mL/min per 1.73 m^2^), who had received dietary counseling and nutritional education for 12 months. The flow chart of patient recruitment is shown in Supplementary Figure S1. A total of 92 patients with stage 4 or 5 CKD were initially enrolled from Chang Gung Memorial Hospital in Chiayi. Patients were excluded if they had (1) unstable medical conditions or active infections such as acute myocardial infarction, acute stroke and pneumonia; (2) decompensated liver diseases; (3) concurrent malignancy; (4) uncontrolled dietary protein intake. Thus, 50 patients were included in the study. Normal controls (n = 15) were defined as subjects who had no history of CKD and an eGFR > 60 mL/min per 1.73 m^2^. During the follow-up period from 2008 to 2016, study outcomes were the onset of ESRD (defined as maintenance dialysis for ≥28 days, or an eGFR < 5 mL/min per 1.73 m^2^ for ≥28 days) or all-cause mortality. Baseline demographic and biochemical data were recorded quarterly. This study was approved by the Institutional Review Board of the Chang Gung Memorial Hospital (IRB #201600845B0C501), and written informed consent was obtained from each participant.

### 4.2. Human Serum and Kidney Samples

Serum samples of healthy and CKD subjects were obtained from Tissue Bank, Chang Gung Memorial Hospital in Chiayi. Kidney samples were obtained from CKD and non-CKD subjects with renal masses suspicious for renal carcinoma. Radical nephrectomy specimens of patients were collected, and then the surrounding unaffected areas of nephrectomy specimens were taken for analysis. This study was approved by the Institutional Review Board of the Chang Gung Memorial Hospital (IRB #201600845B0C501) and all patients provided written informed consent. 

### 4.3. Western Blot Analysis

Western blotting was performed as mentioned previously [46]. Briefly, protein samples were separated in 8–10% SDS-polyacrylamide gel, and then the separated proteins were transferred onto polyvinylidene difluoride (PVDF) membranes (IEVH85R; Millipore, Burlington, MA, USA). The primary antibodies against human or rat DKK1 (sc-25516; Santa Cruz, Dallas, TX, USA), mouse DKK1 (AF1096; R&D Systems, Minneapolis, MN, USA), fibronectin (ab45688; Abcam, Cambridge, UK), TGF-β1 (BS1361; Bioworld Technology, Dublin, OH, USA), α-smooth muscle actin (ab7817; Abcam, Cambridge, UK), β-catenin (#4970S; Cell Signaling, Boston, MA, USA), collagen IV (NB120-6586; Novus, Centennial, CO, US) and actin (#4970S; Cell Signaling, Boston, MA, USA) were used in this study. After removing the unbound primary antibody, the membranes were incubated with suitable secondary antibodies. The resultant antigen-antibody complexes were visualized using the Western Lighting Chemiluminescence Reagent (#NEL105001EA; PerkinElmer, Waltham, MA, USA).

### 4.4. In Vivo CKD Animal Model 

Male Sprague-Dawley rats (BioLasco Biotechnology Co., Taipei, Taiwan), weighing 350 to 380 g, were housed in microisolator cages under a 12-h light/dark cycle, and allowed food and water ad libitum. A total of 24 male rats were randomly divided into four groups (n = 6 per group). All animal protocols were approved by the Chang Gung Institutional Animal Care and Use Committee (no. 2016012903). Rats were anaesthetized with intraperitoneal injection of 6% chloral hydrate (0.5 mL/100 g body weight), and the 5/6-nephrectomy was generated by a nephrectomy of the right kidney and infarction of 2/3 of the left kidney as described previously [47,48]. For the sham group, laparotomy was performed without 5/6 nephrectomy. At 1 week after 5/6-nephrectomy, rats were intraperitoneally injected with the DKK1-sense (DKK1-S) or DKK1-antisense (DKK1-AS) oligonucleotide (40 μg/kg per day) for 8 weeks (5 consecutive days per week). The end-capped phosphorothioate DKK1-S oligonucleotide (5′-TCTGGTCCAAGATCTGAT-3′) and DKK1-AS oligonucleotide (5′-TACAGATCTTGGACCAGA-3′) were obtained from Bio Basic Inc. (Markham, Ontario, Canada). All rats were sacrificed at 9 weeks after surgery. 

### 4.5. Biochemical Assays of Blood and Urine

Serum DKK1 levels in human and rats were measured using Human Dkk-1 Quantikine ELISA kit (#DKK100; R&D Systems, Minneapolis, MN, USA) and Mouse Dkk-1 Quantikine ELISA kit (#MKK100; R&D Systems, Minneapolis, MN, USA), respectively. Levels of blood urea nitrogen (BUN) and serum creatinine (Cr) were determined using MeDiPro BUN kit (#BC-0012; Formosa Bio. Tech., Taipei, Taiwan) and MeDiPro CREA kit (#BC-0017; Formosa Bio. Tech., Taipei, Taiwan), respectively. Levels of urine protein (Autokit Micro TP; Wako Chemicals, Richmond, VA, USA), albumin (Autokit Microalbumin; Wako Chemicals, Richmond, VA, USA) and creatinine (Creatinine assay kit, InnoChem, Gyeonnggi-do, Korea) were analyzed using the DRI-CHEM 4000 Veterinary Chemistry Analyzer, and then the urinary total protein-to-creatinine ratio (TP/Cr) and the urinary albumin-to-creatinine ratio (ACR) were calculated. 

### 4.6. Periodic Acid-Schiff (PAS) Staining, Immunohistochemistry and Immunofluorescence

The periodic acid-Schiff (PAS) staining was performed using the PAS staining kit (#395B-1KT; Sigma-Aldrich, St. Louis, MO, USA). For immunohistochemistry, human kidney sections were stained with the primary antibodies against DKK1 (sc-25516; Santa Cruz, Dallas, TX, USA), fibronectin (ab45688; Abcam, Cambridge, UK) or TGF-β1 (BS1361; Bioworld Technology, Dublin, OH, USA), whereas rat kidney sections were stained with antibodies against DKK1 (sc-25516; Santa Cruz, Dallas, TX, USA), TGF-β1 (E11264; Spring Bioscience, Pleasanton, CA, USA), collagen IV (BSB5355; BioSB, Santa Barbara, CA, USA), fibronectin (BS1644; Bioworld Technology, Dublin, OH, USA) or IL-1β (sc-7884; Santa Cruz, Dallas, TX, USA). For quantification of PAS and immunohistochemical staining, six areas from each section were analyzed using the Image-Pro Plus software (Media Cybernetics, Silver Spring, MD, Version 6.3). Immunofluorescence staining of rat kidney sections with the β-catenin antibody (GTX101435; GeneTex, Irvine, CA, USA) was conducted according to the previously described procedure [49]. The mean fluorescence intensity of β-catenin in kidney sections was quantified using the CellSens software package Version 2.1 (Olympus Medical Systems, Tokyo, Japan).

### 4.7. In Vitro Mesangial Cell Cultures

Mouse mesangial cell line SV40 MES 13 (ATCC; CRL-1927) was routinely cultured in DME/F-12 (3:1 mixture) medium supplemented with 5% fetal bovine serum. In some experiments, mouse mesangial cells were cultured in medium supplemented with human serum, or were treated with anti-DKK1 antibody (#AF1096; R&D Systems, Minneapolis, MN, USA), normal IgG control (#AB-108-C; R&D Systems, Minneapolis, MN, USA), recombinant human DKK1 (5439-DK; R&D Systems, Minneapolis, MN, USA) or recombinant mouse DKK1 (5897-DK; R&D Systems, Minneapolis, MN, USA). 

### 4.8. Quantitative Reverse Transcription-PCR (RT-qPCR)

RT-qPCR was performed as described previously [46]. Briefly, total RNAs from cultured cells or renal glomeruli isolated with laser capture microdissector (VERITAS^TM^; Arcturus Bioscience Inc., Mountain View, CA, USA) were extracted using TRI Reagent (T9424; Sigma-Aldrich, St. Louis, MO, USA). An amount of 1 μg of total RNA from each sample was reversely transcribed into cDNA, and the PCR mixtures (25 μL) that contained cDNA template (equivalent to 20 ng of total RNA), forward and reverse primers (2.5 μM) and 2X iQ^TM^ SYBR Green Supermix were prepared. Nucleotide sequences of PCR primers used in the study are shown in Supplementary Figure S4.

### 4.9. Statistical Analysis

The association of DKK1 level and the risk of outcome (i.e., dialysis and mortality) was evaluated using the Cox proportional hazard model. Several covariates were adjusted in the Cox models, including age, sex, baseline eGFR, hemoglobin, albumin, high-sensitivity C-reactive protein (hs-CRP) and daily urine output. The ability of serum DKK1 level on discriminating dialysis was evaluated using the area under the receiver-operating characteristic curve (AUC). The AUC was calculated using the Harrell’s concordance index in the survival analysis. The baseline characteristics of patients between the low and high subgroups of DKK1 level were compared using t-tests for continuous variables and Fisher’s exact test for categorical variables. The Kaplan–Meier survival rates of dialysis between the low and high subgroups of DKK1 level were compared using log-rank tests. The cutoff point for DKK-1 level in the survival analysis was obtained using R version 3.6.3 (R Development Core Team) and the “survminer” package (Version 0.4.6 updated on 3 September 2019). For in vitro assays and animal experiments, data were presented as means ± standard deviation (SD). A two-sample t-test was utilized to evaluate differences between the control group and experimental group, whereas parametric ANOVA and a Bonferroni post hoc test were utilized to analyze differences among different treated groups. These statistical analyses were conducted using SPSS 25 (IBM SPSS Inc., Chicago, IL, USA), and *p* < 0.05 was considered statistically significant.

## 5. Conclusions

Collectively, we showed that serum levels of DKK1 were positively associated with the fast progression to ESRD in patients with advanced CKD. Furthermore, we demonstrated that DKK1 functioned as a profibrotic mediator in CKD rats and in cultured mesangial cells. These findings strongly suggest that DKK1 may be a useful predictor for CKD progression and a potential therapeutic target of CKD progression.

## Figures and Tables

**Table 1:** Baseline characteristics of advanced CKD patients stratified by the optimal cutoff level of serum DKK1 (1526.4 pg/mL).

Variable	Total (n = 50)	≥1526.4 pg/mL (n = 22)	<1526.4 pg/mL (n = 28)	*p* Value
Male, n (%)	36 (72.0)	13 (59.1)	23 (82.1)	0.113
Age, year	60.7 ± 13.5	63.0 ± 11.1	59.0 ± 15.1	0.308
Body mass index, kg/m^2^	25.2 ± 3.6	25.3 ± 3.1	25.1 ± 4.0	0.814
Diabetes, n (%)	21 (42.0)	10 (45.5)	11 (39.3)	0.775
Hypertension, n (%)	46 (92.0)	19 (86.4)	27 (96.4)	0.308
Baseline eGFR, mL/min/1.73 m^2^	17.1 ± 8.5	16.4 ± 9.0	17.7 ± 8.1	0.614
Albumin, mg/dL	3.67 ± 0.36	3.66 ± 0.41	3.72 ± 0.31	0.515
Creatinine clearance rate, mL/min	24.2 ± 13.5	22.2 ± 12.5	25.7 ± 14.3	0.374
Blood urea nitrogen, mg/dL	47.8 ± 23.6	54.8 ± 27.0	42.3 ± 19.3	0.063
Hemoglobin, g/dL	11.3 ± 2.1	10.8 ± 2.4	11.7 ± 1.8	0.105
hs-CRP, mg/dL	2.0 ± 3.1	2.1 ± 3.1	1.9 ± 3.2	0.838
HbA1c, %	6.1 ± 1.3	6.0 ± 1.0	6.2 ± 1.5	0.608
Daily urine output, mL	2189 ± 645	1944 ± 491	2382 ± 693	0.015
Total protein (urine), mg/day	75.9 ± 88.5	96.0 ± 100.9	60.1 ± 75.6	0.156
Total protein intake, kg	0.84 ± 0.25	0.89 ± 0.22	0.80 ± 0.27	0.196

Abbreviation: eGFR, estimated glomerular filtration rate; hs-CRP, high-sensitivity C-reactive protein; HbA1c, glycated hemoglobin.

**Table 2:** Association of serum DKK1 level (per 100 pg/mL increase) with the risk of outcomes (n = 50).

Outcome/Model	Adjusted HR (95% CI)	*p* Value
Dialysis		
Unadjusted model	1.046 (0.998–1.097)	0.059
Model 1	1.04 (0.99–1.10)	0.145
Model 2	1.07 (1.01–1.15)	0.031
Model 3	1.08 (1.01–1.15)	0.026
Model 4	1.08 (1.01–1.15)	0.035
Mortality		
Unadjusted model	1.05 (0.98–1.12)	0.138
Model 1	1.05 (0.96–1.15)	0.257
Model 2	1.06 (0.96–1.17)	0.233
Model 3	1.06 (0.96–1.17)	0.231
Model 4	1.06 (0.96–1.17)	0.237

Model 1 adjusted for age and sex; Model 2 adjusted for age, sex, baseline eGFR, hemoglobin and albumin; Model 3 adjusted for age, sex, baseline eGFR, hemoglobin, albumin and hs-CRP; Model 4 adjusted for age, sex, baseline eGFR, hemoglobin, albumin, hs-CRP and daily urine output; Abbreviation: HR, hazard ratio; CI, confidence interval; eGFR, estimated glomerular filtration rate; hs-CRP, high-sensitivity C-reactive protein.

## Data Availability

All data presented in the study are available upon request from the corresponding author.

## Supplementary Materials

The supporting information can be downloaded at: https://www.mdpi.com/article/10.3390/ijms24087679/s1.
