# Supplementary material for: Dickkopf-1 Acts as a Profibrotic Mediator in Progressive Chronic Kidney Disease
Source: Int J Mol Sci. 2023 Apr 21;24(8):7679. doi: 10.3390/ijms24087679 (PMC10143456; doi:10.3390/ijms24087679)
Supplement: Supplementary file 1 [file ijms-24-07679-s001.zip › ijms-2324010-supplementary.pdf]

Supplementary Table and Figures

Table S1. Serum DKK1 levels (pg/ml) in healthy controls and CKD patients

Characteristics	Mean ± SD	Median (Range)
Healthy controls (n = 15)	176.2 ± 132.2	166.0 (2.2 to 493.7)
CKD patents (n = 50)	1559.4 ± 701.6	1440.0 (574.8 to 4581.6)
CKD/DKK1-high (n = 22)	2130.8 ± 657.0	1996.8 (1526.4 to 4581.6)
CKD/DKK1-low (n = 28)	1110.6 ± 287.5	1136.4 (574.8 to 1462.8)

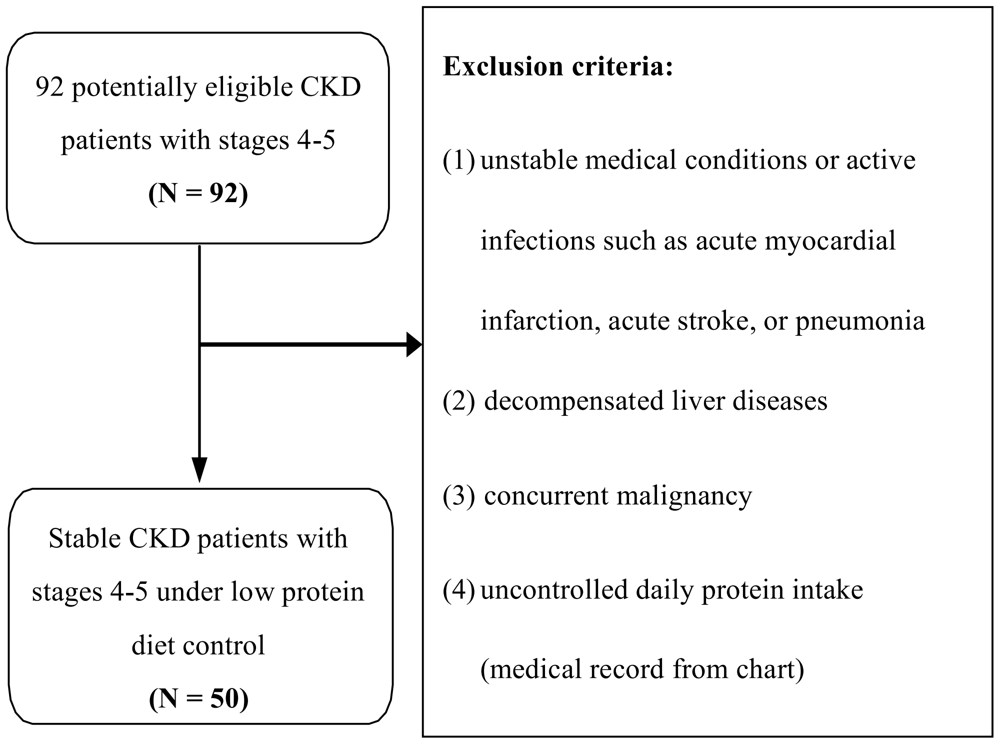

Figure S1. The flow chart of CKD patient recruitment and exclusion.

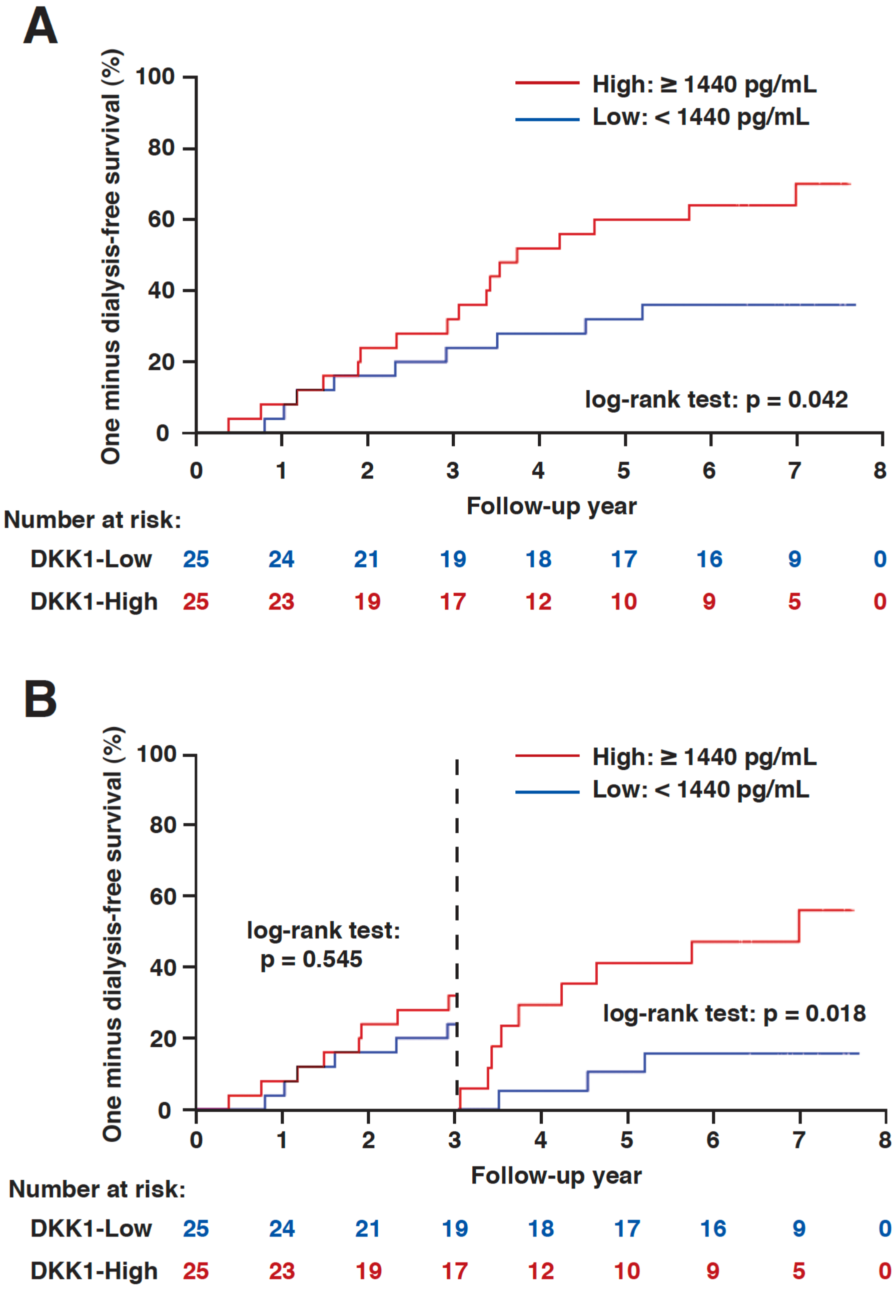

Figure S2. Kaplan-Meier analysis of dialysis-free survival in CKD patients with high or low serum DKK1. (A) One minus dialysis-free survival analysis for the serum DKK1-high (≥ 1440 pg/mL; n = 25) and DKK1-low (< 1440 pg/mL; n = 25) subgroups of CKD patients with stages 4-5 (log-rank test). (B) Stratification by 3-year follow-up in the serum DKK1-high and DKK1-low subgroups.

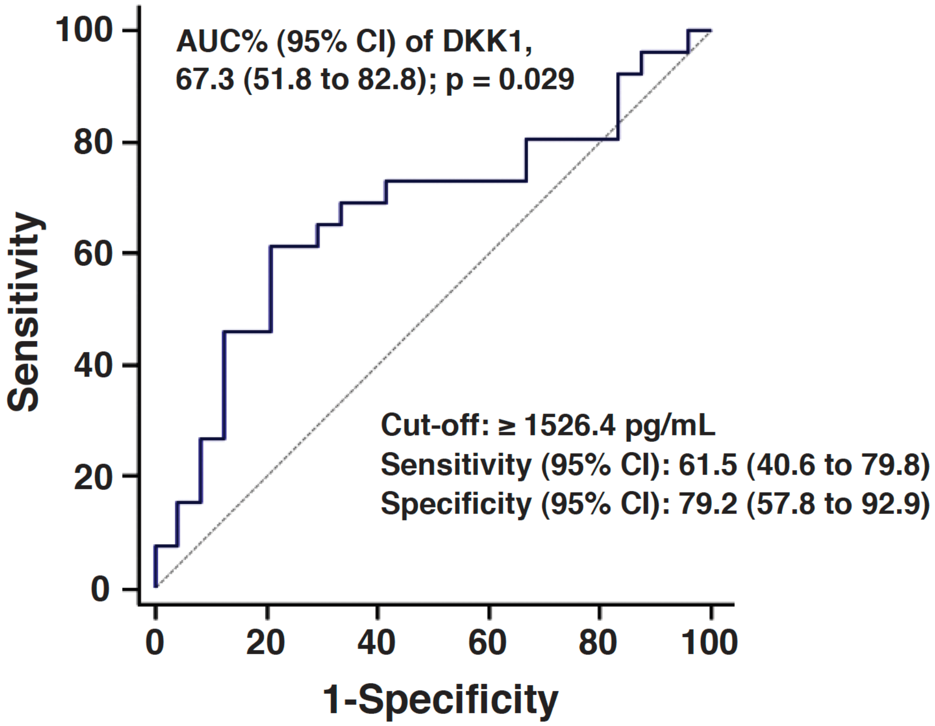

Figure S3. The ROC curve for the optimal cutoff point of serum DKK1 concentration. The most discrimination cutoff level for serum DKK1 was 1526.4 pg/mL with an AUC value of 0.673 ($p = 0.029$).

**Gene name**	**Primer Sequences (5'-3')**	**Product size (bp)**
DKK1 (rat)	TTACTGTGGGGAAGGTCTGG<br>ACATCCTTGGGATTGAGCTG	178
FBN (rat)	GTGGCTGCCTTCAACTTCTC<br>AGTCCTTTAGGGCGGTCAAT	231
TGF-β1 (rat)	TGAGTGGCTGTCTTTTGACG<br>TGGGACTGATCCCATTGATT	154
Actin (rat)	CGCCAACCGCGAGAAGAT<br>CGTCACCGGAGTCCATCA	168
DKK1 (mouse)	TCCGTCTGCCTCCGATCATC<br>GCCTTTCCGTTTGTGCTTGG	123
FBN (mouse)	AATGGAAAAGGGGAATGGAC<br>CTCGGTTGTCCTTCTTGCTC	244
TGF-β1 (mouse)	TTGCTTCAGCTCCACAGAGA<br>TGGTTGTAGAGGGCAAGGAC	183
Actin (mouse)	AGCCATGTACGTAGCCATCC<br>CTCTCAGCTGTGGTGGTGAA	228

Figure S4. Primer sequences for quantitative RT-PCR.
